# Crystalloid Liberal or Vasopressors Early Resuscitation in Sepsis-Study of Treatment’s Echocardiographic Mechanisms (CLOVERS-STEM)

**DOI:** 10.1097/CCE.0000000000001182

**Published:** 2024-12-09

**Authors:** Michael J. Lanspa, Akram Khan, Patrick G. Lyons, Michelle N. Gong, Ali A. Naqvi, Siddharth Dugar, Abhijit Duggal, Nicholas J. Johnson, Jacob H. Schoeneck, Lane Smith, Somnath Bose, Nathan I. Shapiro, Tatyana Shvilkina, Danielle Groat, Jason R. Jacobs, Troy D. Olsen, Steven Cannavina, Daniel B. Knox, Eliotte L. Hirshberg, Wesley H. Self, Samuel M. Brown

**Affiliations:** 1 Intermountain, Critical Care Echocardiography Service, Salt Lake City, UT.; 2 Division of Pulmonary and Critical Care Medicine, Oregon Health & Sciences University, Portland, OR.; 3 Division of Critical Care Medicine, Montefiore Medical Center, New York, NY.; 4 Department of Critical Care, Cleveland Clinic, Cleveland, OH.; 5 Department of Emergency Medicine, University of Washington, Seattle, WA.; 6 Department of Emergency Medicine, Wake Forest Baptist, Winston-Salem, NC.; 7 Department of Emergency Medicine, Beth Israel Deaconess Medical Center, Boston, MA.; 8 Department of Emergency Medicine, Vanderbilt University, Nashville, TN.

**Keywords:** echocardiography, fluid, septic cardiomyopathy, strain, vasopressor

## Abstract

**IMPORTANCE:**

Receipt of fluid and vasopressors, common treatments in septic shock, may affect cardiac function.

**OBJECTIVES:**

We sought to determine whether a liberal or restrictive fluid resuscitation strategy was associated with changes in cardiac function.

**DESIGN:**

We prospectively studied a subset of patients enrolled in the Crystalloid Liberal or Vasopressors Early Resuscitation in Sepsis (CLOVERS) trial, performing echocardiography at baseline and at 24 hours after randomization. Among patients who had an echocardiogram performed at 24 hours, we measured left ventricular global longitudinal strain (LV GLS) and right ventricular free-wall longitudinal strain (RVFWLS). We performed linear regressions with dependent variables of LV GLS, change in LV GLS (ΔLV GLS), and RVFWLS using treatment assignment as an independent variable. We adjusted for ratio of early diastolic mitral inflow velocity to early diastolic mitral annulus velocity, mean arterial pressure, and history of congestive heart failure and myocardial infarction.

**SETTING:**

Emergency department and ICUs.

**PATIENTS:**

Adults with sepsis enrolled in the CLOVERS trial.

**MAIN OUTCOMES AND MEASURES:**

We enrolled 180 patients. Our analytic cohort comprised 131 patients with an echocardiogram performed at 24 hours. We observed no differences between treatment arms with respect to demographic, clinical, or echocardiographic data at baseline. We observed no association between restrictive fluid assignment and LV GLS (coefficient, 1.22; *p* = 0.23), ΔLV GLS (–1.97; *p* = 0.27), or RVFWLS (2.33; *p* = 0.19).

**CONCLUSIONS AND RELEVANCE:**

In a subset of patients enrolled in CLOVERS, we observed no association between receipt of fluid and vasopressors and short-term changes in cardiac function. Decreased enrollment may limit inferences.

**KEY POINTS**

**Question:** Does a liberal or restrictive fluid resuscitation strategy affect short-term cardiac function in sepsis and septic shock?

**Findings:** Although septic myocardial dysfunction was common, we found no association between treatment group assignment and change in cardiac function at 24 hours.

**Meanings:** As the main study was stopped early with no difference in clinical outcomes, it is possible that these resuscitation strategies may not elicit a difference in unselected septic patients or that these markers may be insensitive to these strategies.

Septic shock accounts for 10% of all ICU admissions and 30% of all ICU mortality in the United States (1). Despite recent improvements in mortality for sepsis (2), hospital mortality for patients with septic shock remains 22–50% (3, 4). Patients with septic shock frequently have septic cardiomyopathy, which is characterized by cardiac systolic and diastolic dysfunction, among other findings (5–7). Treatments for septic shock may include administration of IV fluids or vasoactive medications. While these therapies can favorably increase cardiac preload, mean arterial pressure (MAP), or cardiac contractility, excess catecholamines may exert direct and indirect cardiotoxic effects (8, 9). Excess fluid administration could result in worsened interstitial edema and worsened right ventricular (RV) function (6, 10, 11).

The Crystalloid Liberal or Vasopressors Early Resuscitation in Sepsis (CLOVERS) trial randomized patients with sepsis-associated hypotension to one of two protocolized treatments: emphasis on fluid boluses (liberal fluids) or early vasopressor infusions (restrictive fluids) for the first 24 hours of their hospital stay (12). Patients were enrolled primarily in the emergency department after 1–3 L of IV fluid was administered. The CLOVERS study thus provides a unique opportunity to investigate the cardiac effects of these treatments. Specifically, we sought to determine whether a restrictive or liberal fluid strategy resulted in left or RV impairment at 24 hours and whether patients with ventricular dysfunction at enrollment exhibited differential treatment effects or outcomes.

## METHODS

### Study Design and Study Oversight

The CLOVERS-Study of Treatment’s Echocardiographic Mechanisms (CLOVERS-STEM) is an observational ancillary study within the CLOVERS multicenter, randomized, unblinded superiority trial that was funded by the National Institutes of Health/National Heart, Lung, and Blood Institute as part of the Prevention and Early Treatment of Acute Lung Injury Network. The study was approved by the Vanderbilt institutional review board (Study of Treatment’s Echocardiographic Mechanisms, No. 017456, approved April 26, 2019). All study procedures were followed in accordance with the ethical standards of the Vanderbilt institutional review board on human experimentation and with the Helsinki Declaration of 1975. All patients or their legal authorized representatives provided written informed consent for participation in the trial and in the ancillary study.

### Patients

Eligible patients met inclusion criteria for CLOVERS: Adult patients (≥ 18 yr old) with a suspected or confirmed infection (broadly defined as the administration or planned administration of antibiotic agents) and sepsis-induced hypotension (systolic blood pressure, < 100 mm Hg after the administration of ≥ 1000 mL of IV fluid). Patients were excluded if it had been more than 4 hours since meeting inclusion criteria, more than 24 hours since presentation at the hospital, or receipt of more than 3000 mL IV fluid before enrollment. Additionally, patients were excluded if there was presence of fluid overload or severe volume depletion from nonsepsis causes, as part of the CLOVERS criteria. There were no additional inclusion criteria for this ancillary study; the only additional exclusion criterion was an allergy to ultrasound-enhancing agents.

### Study Procedures

Enrolled patients underwent a comprehensive transthoracic echocardiogram at time of enrollment and a second echocardiogram 24 hours later. Serum troponin was also obtained at the time of each echocardiogram. If a full clinical echocardiogram was performed that met the standards of the research echocardiogram within the study time window, that clinical echocardiogram could be substituted for the research echocardiogram. The selected sites for this study (7/60 sites in CLOVERS) routinely perform echocardiography on critically ill patients using sonographers working within an echocardiography laboratory accredited by the Intersocietal Accreditation Commission. Before participating in the study, sites submitted a sample echocardiogram, which was reviewed and approved by the Intermountain Critical Care Echocardiography Core Imaging Laboratory.

### Echocardiographic Assessments

Left ventricular (LV) global longitudinal strain (LV GLS) and RV free-wall longitudinal strain (RVFWLS) were measured using EchoInsight (Epsilon Imaging, Ann Arbor, MI) software (Epsilon Imaging). Other standard echocardiographic parameters were measured using Digisonics Digiview software (Digisonics, Houston, TX). All measurements were performed by a dedicated research cardiac sonographer (T.D.O. or S.C.) and overread by a level II echocardiographer physician (M.J.L. or E.L.H.). In cases where limited image quality prevented accurate assessment of LV GLS, we substituted longitudinal strain from the apical four-chamber only, based on prior precedent (13, 14). RVFWLS was calculated from the apical right-ventricular-focused view if available and standard apical four-chamber if not.

### Additional Clinical Data

We recorded demographic data and medical comorbidities. Additionally, we recorded vital signs, receipt of fluid before randomization, receipt of vasopressors at the time of enrollment, and receipt of mechanical ventilation. Additionally, we calculated Sequential Organ Failure Assessment (SOFA) scores at days 1 and 3 (i.e., the calendar day of CLOVERS enrollment) (15). If patients were discharged alive before day 3, they were assigned a day 3 SOFA value of 0, while those who died before day 3 were assigned the worst SOFA score of 24.

For our primary analyses, we compared 24-hour LV GLS, the difference between baseline LV GLS and 24-hour LV GLS (ΔLV GLS), and the difference between day-1 SOFA and day-3 SOFA (ΔSOFA) between the two treatment groups using multivariable linear regression. We used logistic regression to compare mortality between the treatment groups. The regression models for LV GLS and ΔLV GLS included MAP and cardiac preload (ratio of early diastolic mitral inflow velocity to early diastolic mitral annulus velocity [E/e′]) at the time of the 24-hour echo, as well as prior history of congestive heart failure and myocardial infarction as covariates. Covariates for ΔSOFA and mortality included baseline LV GLS and an interaction term between treatment assignment and baseline LV GLS. In our secondary analysis, we compared the treatment groups with multivariable linear regression with respect to 24-hour RVFWLS. Covariates included cardiac E/e′ at 24 hours and mechanical ventilation. In our tertiary analyses, we focused on heterogeneity of treatment on ΔSOFA by building two multivariable linear regression models with interaction terms. Covariates in both models included 24-hour E/e′ and day-1 SOFA, while one model included an interaction term between treatment group and baseline LV GLS and the other modeled the interaction between treatment group and baseline RVFWLS.

In a sensitivity analysis, we used multiple imputations with chained equations to populate missing data points: E/e′, LV GLS, ΔLV GLS, RVFWLS, and ΔSOFA (16). Additional data points used to inform the imputation included baseline MAP, day 1 SOFA, mechanical ventilation, age, sex, Charlson Comorbidity Index, body mass index, baseline vitals (e.g., heart rate, respiratory rate, temperature), history of arrhythmia, ICU-level care, and in-hospital mortality. In post hoc analyses, we used multivariable linear regression to assess: 1) the impact of including baseline LV GLS as an additional covariate in the primary analysis model for 24-hour LV GLS and 2) the relationship between ΔSOFA and ΔLV GLS, with ΔLV GLS as the independent variable and treatment assignment as a covariate.

The Mann-Whitney *U* test was used to identify differences in unadjusted echocardiogram readings between the treatment arms. We did not correct for multiple comparisons and consider all analyses to be hypothesis generating. A *p* value of less than 0.05 was considered significant. All analyses were performed with R, Version 4.0.3 (Vienna, Austria).

### Power Calculation

We used Monte Carlo simulations (*n* = 1000 iterations each) to estimate power. We used the distribution of LV GLS among septic patients to estimate generating gamma distributions for the two treatment arms (17). The treatment arm gamma distributions were iteratively shifted away from each other in order to determine the minimum difference sufficient to achieve 80% power per the Wilcoxon rank-sum test (α = 0.05). We calculated that 170 patients (*n* = 85 patients per treatment arm on average) would provide 80% power to detect a difference of 2.6 absolute percentage points for LV GLS between the two treatment groups. To achieve this sample size, we planned to enroll 210 patients, with six deaths within the first 24 hours, four missed echocardiograms at 24 hours, and as many as 30 echocardiograms in which measurements were not adequate for the primary analysis. We anticipated that in the event of higher missingness, we would still have 80% power at 75 patients per group (total evaluable *n* = 150) for a difference of 2.8 absolute percentage points.

## RESULTS

**Figure 1.**
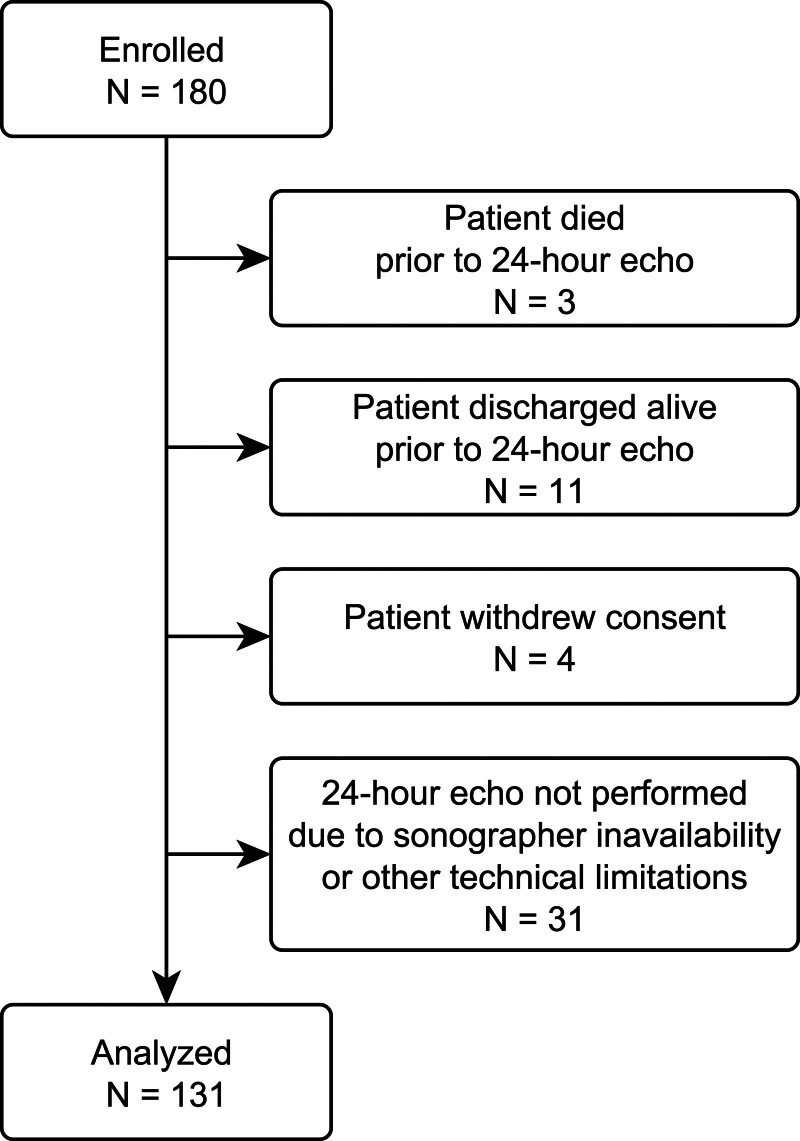
Enrollment of study patients and exclusions leading to final analytic population.

We enrolled 180 patients before the CLOVERS trial was closed on the basis of low conditional power for the primary efficacy endpoint on January 31, 2022. Our analytic cohort was smaller than anticipated, as several patients enrolled in the study later refused echocardiography (*n* = 4) or the study site could not perform the echocardiogram within the study window due to sonographer unavailability. Among performed echoes, we measured LV GLS in 86% of patients. Our final analytic cohort was thus 131 patients (**Fig. 1**). Additionally, several patients (*n* = 52) who were included in the analytic cohort did not have an interpretable echocardiogram at baseline for similar reasons of withdrawing consent, technical limitations, or sonographer unavailability during the study window. Patient characteristics and receipt of fluid and vasopressors are presented in **Supplemental Tables 1** and **3** (http://links.lww.com/CCX/B437).

These patients were representative of the CLOVERS study, with a median age of 59 years, and 44% female. Most (63%) patients were admitted to an ICU, with 20% receiving mechanical ventilation and 22% receiving vasopressors at time of enrollment. They had a median SOFA score of 3 at the time of enrollment. They had received approximately 2 L of fluid before randomization. Twenty-three percent of patients died within 90 days, and 14% were discharged alive before day 3. We observed no significant difference in baseline echocardiographic parameters (**Supplemental Table 2**, http://links.lww.com/CCX/B437).

At 24 hours, we noted no significant difference between treatment arms in unadjusted LV GLS (–17.8 vs. –17.6; *p* = 0.37), ΔLV GLS (0.4 vs. 1.4; *p* = 0.39), or RVFWLS (–19.8 vs. –19.6; *p* = 0.34). In the primary and secondary analyses, we found that treatment assignment was not associated with change in LV GLS, ΔLV GLS, or RVFWLS (**Table 1**). We observed similar non-significant results when we repeated the analyses with imputed missing values. As a post hoc analysis, we repeated the regression for LV GLS, adjusting for baseline LV GLS (**Table 2**), which also demonstrated a non-significant association.

**TABLE 1.** Linear Regressions Evaluating Whether Restrictive Fluid Treatment Is Associated With 24-Hour Left Ventricular Global Longitudinal Strain, Change in Left Ventricular Global Longitudinal Strain, or Right Ventricular Free-Wall Longitudinal Strain

Parameter	Estimate (95% CI)	*p*	*n* (%)
LV GLS			96 (73.3)
Intercept	–20.97		
Restrictive fluid group	1.219 (–0.775 to 3.214)	0.228	
24-hr mean arterial pressure	0.019		
24-hr E/e′	0.139		
Congestive heart failure	6.043		
Myocardial infarction	0.775		
Change in LV GLS			58 (44.3)
Intercept	–1.97		
Restrictive fluid group	1.416 (–1.146 to 3.978)	0.273	
24-hr mean arterial pressure	0.043		
24-hr E/e′	–0.233		
Congestive heart failure	5.002		
Myocardial infarction	4.518		
Right ventricular free-wall longitudinal strain			94 (71.8)
Intercept	–24.69		
Restrictive fluid group	2.332 (–1.196 to 5.860)	0.192	
24-hr E/e′	0.410		
Mechanical ventilation	3.222		

E/e′ = ratio of early diastolic mitral inflow velocity to early diastolic mitral annulus velocity, LV GLS = left ventricular global longitudinal strain.

There was no statistical difference between the randomized groups, liberal fluid vs. restrictive fluid, on LV GLS, change in LV GLS, or right ventricular free-wall longitudinal strain. Similar nonsignificant results were found in a sensitivity analysis when missing values were imputed.

**TABLE 2.** Linear Regression Estimates for and Assignment to Restrictive Fluid Group, Change in Left Ventricular Global Longitudinal Strain, and Adjusting for Baseline Left Ventricular Global Longitudinal Strain

Parameter	Estimate (95% CI)	*p*	*n* (%)
Change in LV GLS			58 (44.3)
Intercept	12.22		
Restrictive fluid group	–0.133 (–2.358 to 2.093)	0.905	
24-hr mean arterial pressure	0.017		
24-hr ratio of early diastolic mitral inflow velocity to early diastolic mitral annulus velocity	–0.375		
Baseline LV GLS	0.555		

LV GLS = left ventricular global longitudinal strain.

We observed no significant association between the interaction of treatment assignment and LV GLS with respect to in-hospital mortality (**Table 3**). We observed no association between the interaction of treatment assignment and baseline LV GLS with respect to ΔSOFA (–0.21; 95% CI, –0.13 to 0.55; *p* = 0.23). We observed no significant associations in related sensitivity analyses with imputed missing data.

**TABLE 3.** Logistic Regression for In-Hospital Mortality With Left Ventricular Global Longitudinal Strain and Assignment of Restrictive Fluid Group

Parameter	OR (95% CI)	*p*	*n* (%)
Hospital mortality			79 (60.3)
Intercept	1.310		
Restrictive fluid group	0.707 (0.013–41.532)	0.863	
LV GLS	1.141 (0.952–1.403)	0.164	
Restrictive fluid group × LV GLS	0.976 (0.753–1.258)	0.847	
Sensitivity analysis (imputed missing values)			
Hospital mortality			131 (100)
Intercept	1.250		
Restrictive fluid group	0.596 (0.027–13.217)	0.741	
LV GLS	1.127 (0.991–1.297)	0.076	
Restrictive fluid group × LV GLS	0.991 (0.813–1.215)	0.930	

LV GLS = left ventricular global longitudinal strain, OR = odds ratio.

## DISCUSSION

This study of patients enrolled in a randomized trial of hemodynamic management for sepsis-induced hypotension found no difference in LV GLS, ΔLV GLS, or RVFWLS with regard to fluid strategy. We did not observe worsened RV function in patients randomized to liberal fluids. Inferences remain limited in our study, as our study cohort was smaller than expected due to early termination of the primary study due to lack of sufficient separation between treatment arms.

The study of septic cardiomyopathy has been challenging (5). Observational studies have been limited by confounding, survivorship bias, immortal time bias, or selection bias (17–19). In these prior studies, observed abnormalities may have been due to treatment differences or may have merely indicated more severe disease. Studying patients enrolled in the CLOVERS trial afforded a unique opportunity. These patients had similar baseline characteristics with protocolized treatment separation. The echocardiograms were performed at prespecified times, allowing for a better understanding of how fluid administration and vasopressors affect cardiac function.

Additionally, most traditional assessments of cardiac function employ ejection fraction or fractional area change, both of which are relatively insensitive to changes in ventricular contractility. Strain directly measures myocardial deformation, outperforms ejection fraction in measuring cardiac contractility, and is more closely associated with mortality in many clinical syndromes (20–22). LV GLS has been demonstrated to be more sensitive than LV ejection fraction for diagnosing LV dysfunction in sepsis (13, 14). The use of strain in our study allows for more sensitive detection of myocardial injury that might be missed with more traditional echocardiographic markers like ejection fraction.

Catecholamine cardiotoxicity can occur when sympathetic overstimulation drives a positive feedback loop of organ dysfunction. Myocardial necrosis has been shown to correlate with the dose and duration of catecholamine therapy (23), and catecholamine use is associated with increased 90-day mortality in septic shock after adjusting for disease severity and propensity to receive catecholamines (9). Septic cardiomyopathy may parallel “stress” cardiomyopathy, in which catecholamines also cause cardiotoxicity and increased mortality (24–26). Under this model of cardiotoxicity, we expected LV strain to be worse among patients assigned to a restrictive fluid/early vasopressor treatment strategy in the CLOVERS study. It is also possible that the increased inotropy of vasopressors may augment strain in healthier hearts. The CLOVERS protocol was designed to fall within the spectrum of usual care for patients with septic shock, and both treatment arms received standard of care therapy for septic shock. It is thus unclear how alternative dosing regimens might affect cardiac function.

Our study did not identify any difference in unadjusted E/e′ between treatment arms. The E/e′ is strongly associated with LV end-diastolic pressure and LV filling pressures (27). In patients with septic shock, E/e′ has been associated with receipt of IV fluid (28). It is possible that the amount of fluid administered was too small to incur a measurable difference in E/e′ at 24 hours. Patients assigned to the liberal fluid arm received 2 L of IV crystalloid as part of the protocol and, for the most part, received very little fluid after that. Isotonic fluid may only remain in the intravascular space for 30–90 minutes (29).

One challenge with interpreting ventricular function in sepsis is that measurements like strain are affected by both disease severity and treatments such as IV fluid and vasopressors, all of which evolve over the course of the disease. Our data are unable to offer useful inferences to determine whether volume, catecholamines, or critical illness might have the greatest impact on ventricular function, specifically strain. Disentangling intrinsic cardiac function from interventions that affect loading conditions is not feasible at present. One should use caution in interpreting abnormal echocardiographic data without incorporating loading conditions. Additionally, our study is limited by the timeframe of the examination. It is possible that our examination at baseline and at 24 hours might miss some of the evolution or resolution of these changes.

We also did not identify changes in RVFWLS between treatment groups. Similar to E/e′, the effect of fluid administration on RV function might not have been measurable at this time point. Although excess fluid may result in interstitial and pulmonary edema, these patients may not have received enough fluid to detect a measurable difference in pulmonary vascular resistance or RV afterload. Although mechanical ventilation may affect assessment of RVFWLS, it was nonsignificant in our analysis.

These observations should be interpreted in the context of the overall findings of the CLOVERS trial, with no clear difference in the prespecified efficacy endpoints. Both therapies remain within standard of care. CLOVERS was a pragmatic trial where clinicians could administer additional fluids or vasopressors to patients in either arm based on clinical judgment. CLOVERS additionally excluded patients who were believed to be profoundly volume depleted or volume overloaded, which might limit generalizability of these findings. These practices could have biased the findings toward the null hypothesis. Despite our findings, catecholamine toxicity does occur in some patients. Over half of our study cohort had abnormal LV GLS, and over half had abnormal RVFWS. Perhaps early echocardiographic assessments might be used to identify these abnormalities and inform treatment strategies. There may be other factors that play a role in cardiac function such as individual patient gene expression and inflammatory response during infection and critical illness. However, at present our study does not suggest a clear association between a liberal fluid or vasopressor predominant resuscitation strategy and short-term changes in cardiac function. Future studies involving sepsis and septic cardiomyopathy might benefit from more detailed assessments of fluid and vasopressor receipt as well as high-quality echocardiography. Additionally, there may be value in identifying the subset of patients with myocardial dysfunction and vasopressor receipt to enrich future studies that might target catecholamine toxicity.

Our study population was lower than planned, limiting the interpretation of a negative result. However, the absolute difference noted in the change of unadjusted LV strain was around 1%, which is below the threshold for a clinically meaningful difference characterized by other studies (30). Additionally, we observed no difference in the average adjusted LV strain between groups at 24 hours. Although we cannot exclude the possibility of a significant association with more patients, we did not observe a trend toward significance.

This study has limitations. The study was stopped early due to the cessation of the parent study, which resulted in lower enrollment than planned. The logistics of performing high-quality, formal echocardiography in critically ill patients proved a limitation at some centers. In addition, the enrollment of patients in the emergency department was associated with higher-than expected rates of refusal of study procedures than was planned. We suspect that this missingness was not completely random, as patients who were able to express a preference to decline an echocardiogram were likely healthier than those who could not. Despite this, we were able to measure strain in 86% of study echocardiograms, which is consistent with other published studies (13).

Although septic myocardial dysfunction was common in this ancillary study of CLOVERS, we found no association between treatment group assignment and change in LV contractility at 24 hours.

## ACKNOWLEDGMENTS

We acknowledge the following individuals for their contributions to this work: Carlos Barbagelata, Mikaele Bown, Jessica Hyde, Genesis Briceno, Jose Pena, Edvinas Pocias, and Jesus Martinez.

## Supplementary Material

## References

[1] Angus DC Linde-Zwirble WT Lidicker J: Epidemiology of severe sepsis in the United States: Analysis of incidence, outcome, and associated costs of care. Crit Care Med 2001; 29:1303–1310 11445675 10.1097/00003246-200107000-00002

[2] Zimmerman JE Kramer AA Knaus WA: Changes in hospital mortality for United States intensive care unit admissions from 1988 to 2012. Crit Care 2013; 17:R81 23622086 10.1186/cc12695 PMC4057290

[3] Shankar-Hari M Phillips GS Levy ML; Sepsis Definitions Task Force: Developing a new definition and assessing new clinical criteria for septic shock: For the third international consensus definitions for sepsis and septic shock (Sepsis-3). JAMA 2016; 315:775–787 26903336 10.1001/jama.2016.0289 PMC4910392

[4] Kaukonen KM Bailey M Suzuki S: Mortality related to severe sepsis and septic shock among critically ill patients in Australia and New Zealand, 2000-2012. JAMA 2014; 311:1308–1316 24638143 10.1001/jama.2014.2637

[5] Beesley SJ Weber G Sarge T: Septic cardiomyopathy. Crit Care Med 2018; 46:625–634 29227368 10.1097/CCM.0000000000002851

[6] Lanspa MJ Cirulis MM Wiley BM: Right ventricular dysfunction in early sepsis and septic shock. Chest 2021; 159:1055–1063 33068615 10.1016/j.chest.2020.09.274 PMC7965651

[7] Lanspa MJ Gutsche AR Wilson EL: Application of a simplified definition of diastolic function in severe sepsis and septic shock. Crit Care 2016; 20:243 27487776 10.1186/s13054-016-1421-3 PMC4973099

[8] Triposkiadis F Karayannis G Giamouzis G: The sympathetic nervous system in heart failure physiology, pathophysiology, and clinical implications. J Am Coll Cardiol 2009; 54:1747–1762 19874988 10.1016/j.jacc.2009.05.015

[9] Wilkman E Kaukonen KM Pettila V: Association between inotrope treatment and 90-day mortality in patients with septic shock. Acta Anaesthe Scand 2013; 57:431–442 10.1111/aas.12056 23298252

[10] Prowle JR Echeverri JE Ligabo EV: Fluid balance and acute kidney injury. Nat Rev Nephrol 2010; 6:107–115 20027192 10.1038/nrneph.2009.213

[11] Vallabhajosyula S Kumar M Pandompatam G: Prognostic impact of isolated right ventricular dysfunction in sepsis and septic shock: An 8-year historical cohort study. Ann Intensive Care 2017; 7:94 28884343 10.1186/s13613-017-0319-9 PMC5589718

[12] Shapiro NI Douglas IS Brower RG; National Heart, Lung, and Blood Institute Prevention and Early Treatment of Acute Lung Injury Clinical Trials Network: Early restrictive or liberal fluid management for sepsis-induced hypotension. N Engl J Med 2023; 388:499–510 36688507 10.1056/NEJMoa2212663 PMC10685906

[13] Lanspa MJ Shahul S Hersh A: Associations among left ventricular systolic function, tachycardia, and cardiac preload in septic patients. Ann Intensive Care. 2017; 7:17 28213737 10.1186/s13613-017-0240-2 PMC5315651

[14] Lanspa MJ Pittman JE Hirshberg EL: Association of left ventricular longitudinal strain with central venous oxygen saturation and serum lactate in patients with early severe sepsis and septic shock. Crit Care 2015; 19:304 26321626 10.1186/s13054-015-1014-6 PMC4553920

[15] Vincent JL Moreno R Takala J: The SOFA (Sepsis-related Organ Failure Assessment) score to describe organ dysfunction/failure. On behalf of the Working Group on Sepsis-Related Problems of the European Society of Intensive Care Medicine. Intensive Care Med 1996; 22:707–710 8844239 10.1007/BF01709751

[16] van Buuren S Groothuis-Oudshoorn K: MICE: Multivariate imputation by chained equations in R. J Stat Softw 2011; 45:1–67

[17] Sanfilippo F Corredor C Fletcher N: Left ventricular systolic function evaluated by strain echocardiography and relationship with mortality in patients with severe sepsis or septic shock: A systematic review and meta-analysis. Crit Care 2018; 22:183 30075792 10.1186/s13054-018-2113-y PMC6091069

[18] Sanfilippo F Corredor C Fletcher N: Diastolic dysfunction and mortality in septic patients: A systematic review and meta-analysis. Intensive Care Med 2015; 41:1004–1013 25800584 10.1007/s00134-015-3748-7

[19] Ehrman RR Sullivan AN Favot MJ: Pathophysiology, echocardiographic evaluation, biomarker findings, and prognostic implications of septic cardiomyopathy: A review of the literature. Crit Care 2018; 22:112 29724231 10.1186/s13054-018-2043-8 PMC5934857

[20] Stanton T Leano R Marwick TH: Prediction of all-cause mortality from global longitudinal speckle strain: Comparison with ejection fraction and wall motion scoring. Circ Cardiovasc Imag 2009; 2:356–364 10.1161/CIRCIMAGING.109.862334 19808623

[21] Kalam K Otahal P Marwick TH: Prognostic implications of global LV dysfunction: A systematic review and meta-analysis of global longitudinal strain and ejection fraction. Heart 2014; 100:1673–1680 24860005 10.1136/heartjnl-2014-305538

[22] Garcia-Montilla R Imam F Miao M: Optimal right heart filling pressure in acute respiratory distress syndrome determined by strain echocardiography. Echocardiogr 2017; 34:851–861 10.1111/echo.13546 28631361

[23] Schmittinger CA Dunser MW Torgersen C: Histologic pathologies of the myocardium in septic shock: A prospective observational study. Shock 2013; 39:329–335 23376953 10.1097/SHK.0b013e318289376b

[24] Margey R Diamond P McCann H: Dobutamine stress echo-induced apical ballooning (Takotsubo) syndrome. Eur J Echocardiogr 2009; 10:395–399 18945727 10.1093/ejechocard/jen292

[25] Templin C Ghadri JR Diekmann J: Clinical features and outcomes of Takotsubo (stress) cardiomyopathy. N Engl J Med 2015; 373:929–938 26332547 10.1056/NEJMoa1406761

[26] Y-Hassan S Settergren M Henareh L: Sepsis-induced myocardial depression and Takotsubo syndrome. Acute Card Care 2014; 16:102–109 24955937 10.3109/17482941.2014.920089

[27] Nagueh SF Middleton KJ Kopelen HA: Doppler tissue imaging: A noninvasive technique for evaluation of left ventricular relaxation and estimation of filling pressures. J Am Coll Cardiol 1997; 30:1527–1533 9362412 10.1016/s0735-1097(97)00344-6

[28] Brown SM Pittman JE Hirshberg EL: Diastolic dysfunction and mortality in early severe sepsis and septic shock: A prospective, observational echocardiography study. Crit Ultrasound J 2012; 4:8 22870900 10.1186/2036-7902-4-8 PMC3512479

[29] Zazzeron L Gattinoni L Caironi P: Role of albumin, starches and gelatins versus crystalloids in volume resuscitation of critically ill patients. Curr Opin Crit Care 2016; 22:428–436 27467273 10.1097/MCC.0000000000000341

[30] Thavendiranathan P Poulin F Lim KD: Use of myocardial strain imaging by echocardiography for the early detection of cardiotoxicity in patients during and after cancer chemotherapy: A systematic review. J Am Coll Cardiol 2014; 63:2751–2768 24703918 10.1016/j.jacc.2014.01.073

