# Supplementary material for: Crystalloid Liberal or Vasopressors Early Resuscitation in Sepsis-Study of Treatment’s Echocardiographic Mechanisms (CLOVERS-STEM)
Source: Crit Care Explor. 2024 Dec 9;6(12):e1182. doi: 10.1097/CCE.0000000000001182 (PMC11631020; doi:10.1097/CCE.0000000000001182)
Supplement: Supplementary file 1 [file cc9-6-e1182-s001.pdf]

Supplemental Table 1: Demographics, baseline characteristics, outcomes (n = 119). Reported as median (IQR) or n (%). No significant differences between groups.

Attribute	Liberal Fluids n=64	Restrictive Fluids n=67	All n=131
Demographics			
Age, years	57.0 (49.0-67.2)	60.0 (43.0-67.0)	59.0 (48.0-67.5)
Female Sex, n (%)	26 (40.6%)	31 (46.3%)	57 (43.5%)
Race, n (%)			
Asian	3 (4.7%)	4 (6.0%)	7 (5.3%)
African American	6 (9.4%)	11 (16.4%)	17 (13%)
White	47 (73.4%)	47 (70.1%)	94 (71.8%)
Unknown	8 (12.5%)	4 (6.0%)	12 (9.2%)
Ethnicity, n (%)			
Hispanic or Latino	9 (14.1%)	8 (11.9%)	17 (13.0%)
Not Hispanic or Latino	52 (81.2%)	55 (82.1%)	107 (81.7%)
Not reported	3 (4.7%)	4 (4.6%)	7 (5.3%)
Baseline			
Charlson comorbidity index	1 (0-3)	1 (0-3)	1 (0-3)
Body mass index	26.5 (23.1-30.6)	25.9 (23.0-31.3)	26.3 (23.1-31.0)
Chronic dialysis, n (%)	5 (7.8%)	3 (4.5%)	8 (6.1%)
Enrollment Vitals			
Mean arterial pressure, mm Hg	66.0 (60.8-72.0)	65.0 (60.0-71.5)	65.5 (60.0-71.9)
Heart rate, min^{-1}	94.5 (72.8-108.0)	94.0 (78.5-106.5)	94.0 (77.0-108.0)
Atrial arrythmia, n (%)	4 (6.2%)	4 (6.0%)	8 (6.1%)
Ventricular arrythmia, n (%)	1 (1.6%)	0 (0%)	1 (0.8%)
Respiratory rate, min^{-1}	19.0 (16.0-22.0)	18.0 (16.0-23.5)	19.0 (16.0-22.5)
Temperature, °C	36.9 (36.6-37.4)	37.0 (36.6-38.0)	37.0 (36.6-37.6)
Acute respiratory distress syndrome, n (%)	0 (0%)	1 (1.5%)	1 (0.8%)
SOFA	3 (1-5)	3 (1-6)	3 (1-5)
Troponin	0.02 (0.00-0.05)	0.02 (0.00-0.09)	0.02 (0.00-0.07)
Enrollment Treatments			
Intensive care unit, n (%)	36 (56.2%)	47 (70.1%)	83 (63.4%)
Mechanical ventilation, n (%)	11 (17.2%)	15 (22.4%)	26 (19.8%)
Vasopressors at enrollment, n (%)	14 (21.9%)	15 (22.4%)	29 (22.1%)
Norepinephrine, n (%)	13 (20.3%)	14 (20.9%)	27 (20.6%)
Vasopressin, n (%)	11 (17.2%)	14 (20.9%)	25 (19.1%)
Total fluids prior to randomization			
Normal saline	2050 (1292-2354)	2011 (1283-2377)	2050 (1285-2355)
Lactated Ringers	0 (0-625)	0 (0-1000)	0 (0-1000)
Plasmalyte	1000 (950-2000)	1000 (0-2000)	1000 (0-2000)

Blood products	238 (98-361)	181 (50-300)	206 (51-348)
Outcomes			
Acute respiratory distress syndrome	1 (1.6%)	2 (3.0%)	3 (2.3%)
Discharged before day 3, n (%)	8 (12.5%)	10 (14.9%)	18 (13.7%)
Mortality before day 3, n (%)	1 (1.6%)	1 (1.5%)	2 (1.5%)
Day 3 SOFA	1.0 (0.0-3.8)	1.0 (0.0-4.0)	1.0 (0.0-4.0)
Day 3 ΔSOFA	1.0 (0.0-3.0)	1.0 (0.0-2.5)	1.0 (0.0-3.0)
Mortality - in hospital, n (%)	10 (15.6%)	8 (11.9%)	18 (13.7%)
Mortality - 90 days, n (%)	14 (21.9%)	16 (23.9%)	30 (22.9%)
Time to mortality, days	8.5 (4.5-18.0)	29.5 (11.0-50.0)	14.5 (5.2-39.8)

Supplemental Table 2: Echocardiographic data. Reported as median (IQR). No significant differences between groups.

Attribute	Liberal Fluids n=64	Restrictive Fluids n=67	All n=131
Baseline Echo Data (N= 79)			
LV EF, %	59.4 (53.6-66.8)	60.8 (55.8-67.6)	60.3 (54.8-67.4)
Left ventricular GLS, %	-19.1 (-22.5--13.9)	-16.2 (-20.2--12.8)	-17.3 (-21.4--13.1)
E/e’	7.7 (6.3-11.9)	7.6 (6.6-10.6)	7.6 (6.5-10.9)
RV FAC, %	35.4 (24.9-41.2)	35.6 (32.4-42.9)	35.5 (29.1-42.9)
RV TAPSE, cm	1.9 (1.6-2.2)	2.1 (1.8-2.3)	2.0 (1.6-2.3)
Right Ventricular Free Wall GLS, %	-18.2 (-21.9--12.0)	-17.7 (-21.5--10.0)	-18.2 (-21.6--10.9)
24-hour Echo Data			
LV EF, %	58.4 (53.5-64.8)	60.3 (54.8-69.7)	59.7 (54.4-67.2)
Left ventricular GLS, %	-17.8 (-21.4--14.5)	-17.6 (-19.9--13.7)	-17.8 (-20.6--14.4)
ΔLeft ventricular GLS, %	0.4 (-4.3-2.2)	1.4 (-2.5-3.9)	0.6 (-3.5-3.4)
E/e’	8.2 (6.9-11.6)	7.6 (6.7-10.4)	8.0 (6.7-11.2)
RV FAC, %	39.8 (26.0-46.5)	32.4 (26.4-41.2)	36.1 (25.9-43.1)
RV TAPSE, cm	-19.8 (-26.2--15.3)	-19.6 (-24.6--12.7)	-19.7 (-25.3--14.2)
Right Ventricular Free Wall GLS, %	-19.8 (-26.2--15.3)	-19.6 (-24.6--12.7)	-19.7 (-25.3--14.2)

Supplemental Table 3: Fluid and vasopressor receipt between treatment arms.Echocardiographic data. Reported as median (IQR). No significant differences between groups.

Attribute	Liberal Fluids n=64	Restrictive Fluids n=67	All n=131	P value
Vasopressor receipt 0 to 24 hours, n (%)	23 (35.9%)	41 (61.2%)	64 (48.9%)	0.007
Vasopressor receipt 48h to day 28, n (%)	35 (20.3%)	24 (13.7%)	59 (17%)	0.046
Received 2L per protocol, n (%)	38 (59.4%)	0 (0%)	38 (29.0%)	<0.001
IV Fluid first 6 Hours, mL	2150 (1650-2525)	598 (202-1140)	1250 (550-2226)	<0.001
IV Fluid first 6 Hours, mL/kg	27.3 (20.6-37.0)	6.0 (2.6-14.2)	16.7 (5.2-29.2)	<0.001
Total volume 6-24 hours	0 (0-2000)	0 (0-138)	0 (0-750)	0.436
